# Supplementary material for: The Systems Biology Research Tool: evolvable open-source software
Source: BMC Syst Biol. 2008 Jun 29;2:55. doi: 10.1186/1752-0509-2-55 (PMC2446383; doi:10.1186/1752-0509-2-55)
Supplement: Additional file 1 — SBRT Archive. An archive of the current version of the Systems Biology Research Tool. [file 1752-0509-2-55-S1.zip › sbrt-1.4.0/doc/developers_guide/api/sbrt/shell/io/fba/package-tree.html]

sbrt.shell.io.fba Class Hierarchy


|  |  |  |
| --- | --- | --- |
| **Overview** \| **Package** \| Class \| Use \| **Tree** \| **Deprecated** \| **Index** \| **Help** | |  |
| **PREV**   **NEXT** | **FRAMES**    **NO FRAMES**     **All Classes** |  |


---


## Hierarchy For Package sbrt.shell.io.fba

**Package Hierarchies:**: All Packages

---

## Class Hierarchy

- java.lang.Object
  - sbrt.shell.io.fba.**CofoofSource** (implements java.util.Iterator<E>)- sbrt.shell.io.fba.**ColumnOrFbaOptOutputFile** (implements sbrt.shell.io.fba.FbaOptOutputFile)- sbrt.shell.io.fba.**FluxCapInputFile** (implements sbrt.shell.io.MapTextInputFile<K,V>)- sbrt.shell.io.fba.**FluxIntervalInputFile** (implements sbrt.shell.io.InputFile<E>)- sbrt.shell.io.fba.**RofoofSource** (implements java.util.Iterator<E>)- sbrt.shell.io.fba.**RowOrFbaOptOutputFile** (implements sbrt.shell.io.fba.FbaOptOutputFile)- sbrt.shell.io.fba.**SerFbaOptOutputFile** (implements sbrt.shell.io.fba.FbaOptOutputFile)- sbrt.shell.io.fba.**SfoofSource** (implements java.util.Iterator<E>)- sbrt.shell.io.**TextInputFile**<E> (implements sbrt.shell.io.InputFile<E>)
                    - sbrt.shell.io.fba.**RxnNamExprSetInputFile**<E,S>

## Interface Hierarchy

- sbrt.kernel.io.**ResultsManager**<T>
  - sbrt.shell.io.**OutputFile**<T> (also extends sbrt.shell.io.SbrtFile)
    - sbrt.shell.io.fba.**FbaOptOutputFile**
  - sbrt.shell.io.**SbrtFile**
    - sbrt.shell.io.**OutputFile**<T> (also extends sbrt.kernel.io.ResultsManager<T>)
      - sbrt.shell.io.fba.**FbaOptOutputFile**

---


|  |  |  |
| --- | --- | --- |
| **Overview** \| **Package** \| Class \| Use \| **Tree** \| **Deprecated** \| **Index** \| **Help** | |  |
| **PREV**   **NEXT** | **FRAMES**    **NO FRAMES**     **All Classes** |  |


---
